# Brain connectivity study of brain tumor patients using MR-PET data: preliminary results

**DOI:** 10.1186/2197-7364-2-S1-A75

**Published:** 2015-05-18

**Authors:** Ana Carina Mendes, Andre Santos Ribeiro, Ana Maria Oros-Peusquens, Karl Josef Langen, Jon Shah, Hugo Alexandre Ferreira

**Affiliations:** Institute of Biophysics and Biomedical Engineering, Faculty of Sciences of the University of Lisbon, Portugal; Centre for Neuropsychopharmacology, Division of Brain Sciences, Department of Medicine, Imperial College London, London, UK; Institute of Neuroscience and Medicine - 4, Forschungszentrum Juelich, Germany

Brain activity results from anatomical and functional connections that can be disrupted or altered due to trauma or lesion. This work presents a first approach on the study of whole-brain connectivity of brain tumor patients using the Multimodal Imaging Brain Connectivity (MIBCA) toolbox. Two patients with glioblastoma lesions located in the left hemisphere (one in the motor cortex and the other in the temporal lobe) underwent simultaneous MRI and dynamic PET scans using a 3T MRI scanner with a BrainPET insert. The following data was acquired: T1-w MPRAGE (1x1x1mm^3^), DTI (dir=30, b=0,800s/mm2, 2x2x2mm^3^), and dynamic 18F-FET PET. The MIBCA toolbox was used to automatically pre-process MRI-PET data and to derive imaging and connectivity metrics from the multimodal data. Computed metrics included: cortical thickness from T1-w data; mean diffusivity (MD), fractional anisotropy (FA), node degree, clustering coefficient and pairwise ROI fibre tracking (structural connectivity) from DTI data; and standardized uptake value (SUV) from PET data. For all the metrics, the differences between left and right hemispherical structures were obtained, followed by a 25% threshold (except for SUV thresholded at 15%). Data was visualized in a connectogram, and both structural connectivity and metrics were studied in regions surrounding lesions. Preliminary results showed increased SUV values in regions surrounding the tumor for both patients. Patients also showed changes in structural connectivity involving these regions and also other more spatially distant regions such as the putamen and the pallidum, including decreased number of fibers between the subcortical structures themselves and with frontal regions. These findings suggest that the presence of a tumor may alter both local and more distant structural connections. Presently, a larger patient sample is being studied along with the inclusion of a control group to test the consistency of the findings.

